# Exposures and Suspected Intoxications to Pharmacological and Non-Pharmacological Agents in Children Aged 0–14 Years: Real-World Data from an Italian Reference Poison Control Centre †

**DOI:** 10.3390/jcm12010352

**Published:** 2023-01-02

**Authors:** Valentina Brilli, Giada Crescioli, Andrea Missanelli, Cecilia Lanzi, Massimo Trombini, Alessandra Ieri, Francesco Gambassi, Alfredo Vannacci, Guido Mannaioni, Niccolò Lombardi

**Affiliations:** 1 Department of Neurosciences, Psychology, Drug Research and Child Health, Section of Pharmacology and Toxicology, University of Florence, 50139 Florence, Italy; 2 Tuscan Regional Centre of Pharmacovigilance, 50122 Florence, Italy; 3 Toxicology Unit, Poison Control Center, Careggi University Hospital, 50134 Florence, Italy

**Keywords:** clinical toxicology, intoxication, poison control centre, clinical practice, children, emergency department, hospitalisation

## Abstract

This study describes the exposures and suspected intoxications in children (0–14 years) managed by an Italian reference poison control center (PCC). A seven-year observational retrospective study was performed on the medical records of the Toxicology Unit and PCC, Careggi University Hospital, Florence (Italy). During the study period (2015–2021), a total of 27,212 phone call consultations were managed by the PCC, of which 11,996 (44%) involved subjects aged 0–14 years. Most cases occurred in males (54%) aged 1–5 years (73.8%), mainly at home (97.4%), and with an oral route of intoxication (93%). Cases mainly occurred involuntarily. Consultations were generally requested by caregivers; however, in the age group 12–14 years, 70% were requested by healthcare professionals due to voluntary intoxications. Cleaners (19.44%) and household products (10.90%) were the most represented suspected agents. Pharmacological agents accounted for 28.80% of exposures. Covariates associated with a higher risk of emergency department visit or hospitalization were voluntary intoxication (OR 29.18 [11.76–72.38]), inhalation route (OR 1.87 [1.09–3.23]), and pharmacological agents (OR 1.34 [1.23–1.46]), particularly central nervous system medications. Overall, consultations do not burden national and regional healthcare facilities, revealing the activity of PCCs as having a strategic role in reducing public health spending, even during the COVID-19 pandemic.

## 1. Introduction

Acute pediatric intoxication is a common and preventable cause of morbidity and mortality worldwide. Exposure to toxic substances can occur at all stages of the child’s development and in different ways, depending on age, environmental factors, and the characteristics of various toxic substances [1].

A study conducted by the Pediatric Emergency Research Networks Poisoning Working Group, which included children younger than 18 years with acute poisonings presenting to 105 emergency departments (EDs) in 20 countries (8 different global regions), showed that 0.47% of pediatric ED admissions were due to intoxications, occurring at home for the most part (80.6%) and with a bimodal peak age distribution [2]. According to the 38th annual report of the American Association of Poison Control Centers’ National Poison Data System, during 2020, more than two million human exposure cases were reported, of which 41.7% involved children ≤5 years [3]. In this age class, the top five most common exposures were cosmetics/personal care products (11.8%), household cleaning substances (11.3%), analgesics (7.57%), foreign bodies/toys/miscellaneous (6.71%), and dietary supplements/herbals/homeopathic (6.44%). Overall, the report showed that medical information requests had shown a 32.6 fold increase, reflecting COVID-19 pandemic calls to poison control centers (PCCs).

Data on large populations are relatively lacking in the literature, particularly in Europe; therefore, it is difficult to estimate the actual incidence of toxic exposure in children. Recently, three retrospective studies on pediatric poisoning were published in Italy. A six-year retrospective study by Berta et al. (2020) showed that 72.9% of cases of pediatric acute intoxication occurred in children <5 years, 85% of intoxications were unintentional, and non-pharmaceutical agents accounted for 59% of cases [4]. A three-year retrospective study by Marano et al. (2021) on Pediatric Poison Control Centre registry data showed that 74.1% of cases involved children aged 1–5 years, 98.2% of intoxications were accidental, and the causative agents were non-pharmaceutical products in 66.7% of cases [5].

In light of this, the aims of this study were to describe the demographics and clinical characteristics of exposures and suspected pediatric (0–14 years) intoxications through the analysis of data collected by an Italian reference PCC and to highlight the role of clinical toxicologists in the early evaluation/management of suspected intoxication in this subset.

## 2. Materials and Methods

This study was performed on data retrieved during phone call consultations by the Toxicology Unit and PCC of Careggi University Hospital, Florence (Italy), from 1 January 2015 to 31 December 2021. Data concerning exposures and probable or acute intoxications from any causative agent in subjects aged 0–14 years, from private individuals or health professionals, were included in the analysis.

For each phone call counselling (first contact), after obtaining the patient’s parents or caregivers’ informed consent to participate in the study, trained clinical toxicologists collected the following information: (1) patient’s demographic characteristics (age, sex); (2) number and characteristics of suspected toxic agents; (3) the place of intoxication occurrence (home, public closed places, open environment/outdoors); (4) description of the event and its circumstances (accidental/involuntary exposure, voluntary intoxication, secondary effect); (5) intoxication route; and (6) symptomatology.

Toxic agents were categorized as follows: pharmacological agents (classified according to the anatomical therapeutic chemical (ATC) classification system); food toxins; animal poisons; mushrooms; plant poisons; CO and other gases; non-pharmacological agents (pesticides; cosmetics and personal hygiene products; substances of abuse; industrial products; other chemical compounds; household products). Household products were further classified into the following subgroups: detergents; hand-wash detergents; laundry detergents; other cleaners; bleaches; caustics; glues; fertilizers; thermometer liquids; toys; button batteries; silica gel; chlorine vapors; varnishes.

A toxicological evaluation was then performed, in order to calculate the poisoning severity score (PSS) [6] (PSS score 0–NONE: no signs or symptoms related to exposure; PSS score 1–MINOR: mild, transient symptoms with spontaneous resolution; PSS score 2–MODERATE: evident or prolonged symptoms; PSS score 3–SEVERE: severe or life-threatening symptoms; PSS score 4–FATAL: death), and to define the plausibility of the intoxication. Intoxications were defined as “absent” (no intoxication), “doubtful”, “possible”, and “confirmed”. Toxicologists could also judge the patient’s symptoms as “independent from the intoxication”.

The phone call consultations were grouped according to the age of the patients (<1 years old; 1–5 years old; 6–10 years old; 11–14 years old), and divided in two cohorts, depending on whether the caller was a private individual or a healthcare professional.

The compilation, archiving of electronic folders, and data extrapolation were carried out using the “ARCHICAV” electronic registry version 1.0. Data were described as numbers and percentages and compared using a Chi-squared test. Multivariate logistic regression (crude and adjusted models) was performed to estimate the odds ratios (ORs) of ED visit or hospitalization according to subjects’ demographic and clinical characteristics (sex, age, number of toxic agents, circumstances, toxic agents, route of exposure) and the most frequently reported pharmacological drug classes.

Statistical significance was considered with a *p*-value ≤ 0.05. Statistical analyses were carried out using Stata 17 (StataCorp).

## 3. Results

During the study period (2015–2021 years), a total of 27,212 phone call consultations were managed by our PCC, of which 11,996 (44.08%) involved subjects aged 0–14 years (Table 1). The majority of exposures and suspected intoxications occurred in males (53.98%) aged 1–5 years (N = 8854). Among all age groups, the most frequently reported route of exposure was the oral route, involving 92.87% of subjects aged 0–11 months, 93.51% of those aged 1–5 years, 87.04% of those aged 6–11 years, and 86.62% of those 12–14 years. The majority of consultations were requested by caregivers; however, in the age group 12–14 years, 70.28% of consultations were requested by healthcare professionals. More than 90% of all exposures and suspected intoxications took place in a domestic environment, through an involuntary means, with a mean time from exposure to consultation that ranged from 2.76 to 6.13 h. Of interest, the majority of voluntary intoxications occurred in the age group 12–14 years (23.14%). Overall, most subjects reported no symptoms at the moment of phone call consultation, followed by those reporting gastrointestinal and neurological symptoms. Among all consultations (N = 11,996), clinical toxicologists advised home observation in 68.58% of case (N = 8228), ED visit in 8.81% (N = 1057), ED observation in 15.04% (N = 1805), and hospitalization in 7.55% (N = 906). A total of 3358 (27.99%) pediatric subjects needed a pharmacological treatment, in particular a symptomatic therapy was prescribed in 22.09% (N = 2651), followed by decontamination in 5.06% (N = 607), and antidotal therapy in 0.83% (N = 100). Notably, none of the exposures and suspected intoxications resulted in the patient’s death.

As reported in Table 2, the majority of subjects were exposed to only one suspected toxic agent (N = 11,833, 98.64%). Overall, the exposures and suspected intoxications managed by our PCC regarded non-pharmacological toxic agents in 68.40% (N = 8206) of cases. Among this subset, cleaners (19.44%) and other household products (10.90%), toys (9.26%), cosmetics (8.15%), and plant poisons (7.18%) were the most represented agents involved in the suspected intoxications. On the other hand, pharmacological agents accounted for 28.80% (N = 3785) of the total suspected intoxications, involving analgesics in 3.39% of cases, antibacterials for systemic use in 2.30%, NSAIDs and antirheumatic products in 2.30%, and psycholeptics in 2.02%, and thyroid therapy in 1.63%. Another class of suspected agents with therapeutic potential, i.e., products belonging to complementary and alternative medicine (CAM), accounted for 2.71% (N = 326) of exposures and suspected intoxications.

The multivariate logistic regression analysis showed that the risk of ED visit or hospitalization was lower for females (adjusted OR 0.88 [95% CI: 0.81–0.95]), and this increased with age, ranging from 1.27 (1–5 years) to 2.52 (12–14 years) (Table 3). Furthermore, in the adjusted models, the analysis showed that the exposure to more than one suspected agent (OR 3.79 [95% CI: 2.65–5.42]), voluntary intoxication (OR 29.18 [95% CI: 11.76–72.38]), pharmacological agents (OR 1.34 [95% CI: 1.23–1.46]), concomitant exposure to pharmacological and non-pharmacological agents (OR 2.03 [95% CI: 0.20–20.13]), and inhalation as route of exposure (OR 1.87 [95% CI: 1.09–3.23]) were the covariates associated with a higher risk of ED visit or hospitalization.

Considering the suspected pharmacological agents (Table 4), the multivariate logistic regression analysis showed that beta blocking agents (OR 10.99 [95% CI: 5.38–22.44]), psycholeptics (OR 4.25 [95% CI: 3.13–5.78]), agents acting on the renin–angiotensin system (OR 4.13 [95% CI: 2.69–6.34]), psychoanaleptics (OR 3.68 [95% CI: 2.60–5.20]), and antiepileptics (OR 2.08 [95% CI: 1.40–3.07]) were the classes associated with a higher risk of ED visit or hospitalization observed in our cohort.

**Figure 1:**
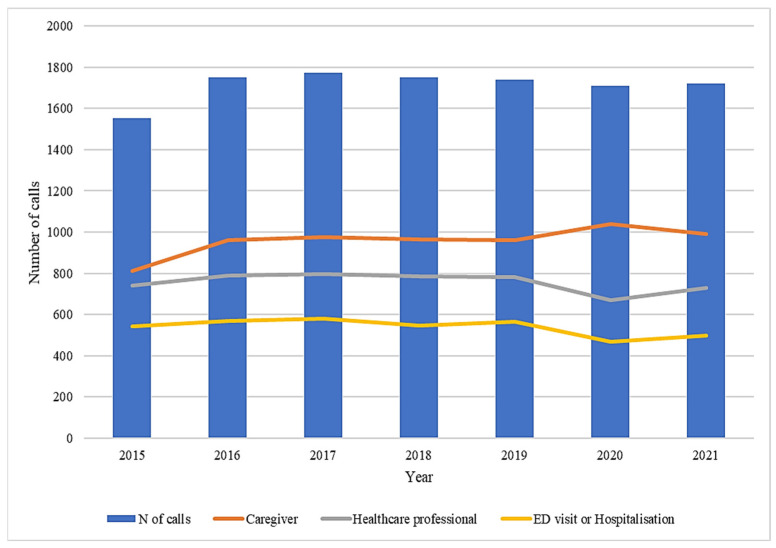
Distribution of calls, qualification and ED visits or hospitalizations during the study period (years 2015–2021).

During the seven-year study period, a consistent number of phone call consultations were observed, ranging from approximately 1600 to 1800 each year (Figure 1).

**Figure 2:**
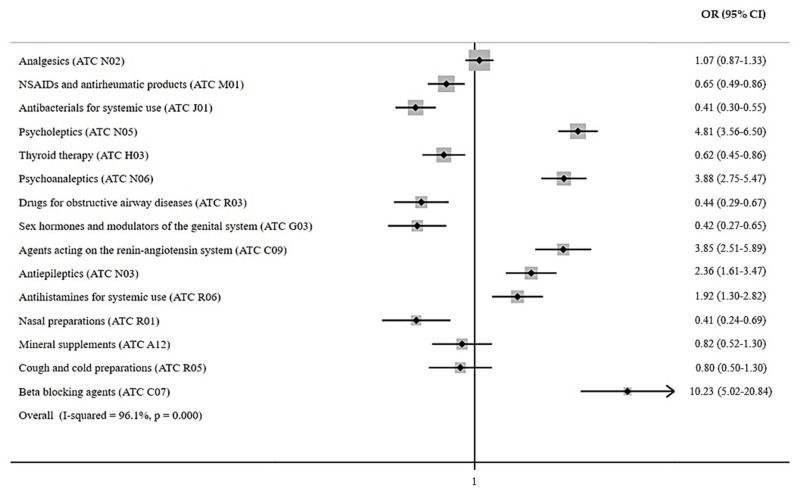
Risk of ED visit or hospitalization according to the most frequently reported pharmacological drug classes.

Considering the pharmacological drug classes involved in the exposures and suspected intoxications in our cohort (Figure 2), a statistically significant risk of an ED visit or hospitalization was observed for the following ATC classes: beta blocking agents (OR 10.23 [95% CI: 5.02–20.84]), psycholeptics (OR 4.81 [95% CI: 3.56–6.50]), psychoanaleptics (OR 3.88 [95% CI: 2.75–5.47]), agents acting on the renin–angiotensin system (OR 3.85 [95% CI: 2.51–5.89]), antiepileptics (OR 2.36 [95% CI: 1.61–3.47]), and antihistamines for systemic use (OR 1.92 [95% CI: 1.30–2.82]).

Although there was no statistically significant difference, exposures and suspected intoxications due to both pharmacological and non-pharmacological agents presented the shortest mean time to clinical toxicology consultation (Table S1). Additionally, caregivers consulted the PCC approximately one hour earlier than healthcare professionals.

Finally, the majority of phone call consultations requested by both caregivers (N = 6703) and healthcare professionals (N = 5293) were due to exposures and suspected intoxications associated with non-pharmacological agents (71.59% and 64.37%, respectively) (Table S2).

## 4. Discussion

Exposures and suspected intoxications in children are a frequent cause of ED visit and hospitalization worldwide; however, there is a lack of information on this preventable public health issue and on the associated potentially life-threatening clinical conditions, particularly in frail subsets. The present epidemiological analysis from a reference PCC in central Italy gives real-world insight into the main demographic, clinical, and pharmaco-toxicological characteristics of intoxications in subjects aged 0–14 before and the during COVID-19 pandemic. To date, this retrospective analysis is the only one conducted in Italy on a representative sample of children over a study period of 7 years. It is relevant that all consultations described here were handled exclusively by clinical toxicology specialists. Furthermore, this study represents the extended epidemiological investigation that followed our preliminary study performed in the general population during the COVID-19 pandemic [7].

In the last two years, particularly in the context of COVID-19 pandemic, several observational studies evaluating the impact of suspected exposures and intoxications in children have been conducted.

Considering that real-world data about acute poisoning in Italian pediatric patients are lacking, different clinical research groups have recently reported new insights on this topic [4,5,8]. The majority of these observational investigations were conducted by pediatricians or other healthcare professionals (i.e., anesthesiologists) working in a pediatric ED, often equipped with a dedicated PCC. On the contrary, our analysis was performed only on the clinical activity of a medical toxicology unit and PCC, where specialists in clinical pharmacology and toxicology probably have the best expertise, in terms of the management of exposures and suspected intoxications in the general population, including children.

In 2020, Berta et al. performed a six-year retrospective evaluation of the Children’s Emergency Department database at the Regina Margherita Hospital of Turin (Italy), where 1030 children under age 14 were accepted with a diagnosis of acute intoxication [4]. In their study, similarly to what was observed in our analysis, the majority of patients were male, with events occurring mostly in children aged 1–4 years, while 59% of patients were exposed to non-pharmaceutical agents. Among these, household cleaning products were the more frequently (49%) reported suspected agents. Regarding exposures to pharmaceuticals, the most frequently involved agents were analgesics (20.8%), psychotropics (18.2%), and cardiovascular (12.6%) drugs. Likewise, in our cohort, analgesics represented the main pharmacological class involved in exposures and suspected intoxications in children, thus indicating the need for additional monitoring of the appropriate use of these medications in clinical practice, especially in the domestic environment. Furthermore, Berta et al. found that 85% of intoxications occurred accidentally, 10.6% as therapeutic error, 2.3% as suicide attempts, and 1.5% for recreational purposes. Additionally, in our sample, the majority of cases were associated with an involuntary toxic exposure, underlining the need to both better store potentially dangerous products, which should always be kept out of reach of children, and to monitor them closely at home, especially with those aged 1–5.

Another study, published in 2021, aimed to describe the characteristics of a large pediatric cohort exposed to xenobiotics, through the analysis of a pediatric PCC registry [5]. This study, conducted in the Pediatric Hospital Bambino Gesù of Rome (Italy), a reference national pediatric hospital, collected data of children whose parents or caregivers contacted the pediatric PCC by phone or who presented to the ED, during a 3 year period (2014–2016). The authors collected data of a total of 2686 children, exposed to both pharmaceutical and non-pharmaceutical agents. Correspondingly to what we observed in our analysis, the authors found that pharmaceutical agent exposure increased with age and that the most common route of exposure was oral with gastrointestinal symptoms. Intentional exposure (substance abuse and suicide attempt), was associated with older age and was comparable to our results.

In 2022, Soave et al. identified risk factors associated with pediatric acute poisoning and proposed prevention strategies for children admitted to ED [8]. They performed a retrospective study in a tertiary care hospital, describing data of 436 children admitted for acute poisoning. In this analysis, the age group 1–5 and male sex were the most represented, with a higher frequency of unintentional poisoning and drug ingestion as the leading cause of intoxication.

International differences in the epidemiology of acute poisonings in children may help to improve prevention. In light of this, in 2019, the Pediatric Emergency Research Networks (PERN) Poisoning Working Group sought to evaluate the international epidemiological differences in acute poisonings in children presenting to EDs from different global regions, including eight different Italian pediatric hospitals [2]. Mintegi et al. conducted this international multicenter cross-sectional prospective study, including children younger than 18 years old with acute poisonings, presenting to a total of 105 EDs in 20 countries, and with a mean follow-up of 1 year. During the study period, 363,245 pediatric ED presentations were registered, of which 1727 were for poisoning, with a significant variation in incidence between the regions. Most poisonings (80.6%) occurred at home with either ingestion (89.0%) or inhalation (7.6%) as the route of exposure. Unintentional exposures accounted for 68.5% of poisonings, with pharmaceuticals (42.7%), household products (26.8%), and pesticides (5.1%) being the most common toxic agents. Furthermore, suicide attempts accounted for 13.8% of total exposures, with pharmaceuticals, mainly psychotropics and acetaminophen, being the most common suspected agents.

Overseas, Desai et al. characterized trends in clinically significant toxic exposures and their management, performing a retrospective review of patients 18 years or younger in the American College of Medical Toxicology’s Toxicology Investigators Consortium (ToxIC) Registry, a self-reporting database completed by bedside consulting medical toxicologists [9]. From 2010 to 2015, the authors analyzed 11,616 cases reported in their registry. Unlike what has been observed in Europe, exposures were most commonly reported in females (57.8%) and adolescents (59.4%). Moreover, intentional ingestions (55.5%) comprised the majority of cases. The most frequent agents of exposure were analgesics (21.0%), and 0.9% of cases resulted in death.

Of interest, the 38th Annual Report of the American Association of Poison Control Centers’ (AAPCC) National Poison Data System (NPDS) [3] reported that children younger than 3 years of age were involved in 30.3% of exposures and children ≤5 years accounted for 41.7% of human exposures in 2020. A prevalence of males was found among cases involving children ≤12 years, but this sex-based distribution was reversed in teenagers and adults. Overall, unintentional exposures outnumbered intentional ones, including children ≤5 years (57.14%). Moreover, the substance categories most frequently involved in pediatric (≤5 years) exposures were cosmetics/personal care products (11.82%), household products (11.30%), analgesics (7.57%), foreign bodies/toys (6.71%), and dietary supplements/herbals/homeopathic products (6.44%). Although children ≤5 years were involved in the majority of exposures, they comprised only 1.27% of fatalities.

From a pharmacological point of view, a result that should not be underestimated regards drugs with action on the central nervous system (CNS). In fact, our study highlighted that most of the ATC classes involved in exposures and suspected intoxications concerned drugs belonging to ATC class N (i.e., analgesics, psycholeptics, psychoanaleptics, and antiepileptics). This is a clinically relevant issue, since several studies have indicated a potential association between parental/caregiver CNS disorders and child mortality [10], particularly an increased risk of child poisoning, burns, and fractures before 5 years of age [11,12]. Our findings, combined with those described by other authors, emphasize the importance of specific interventions for parents/caregivers with CNS disorders. In this context, the clinical pharmacologist and toxicologist operating in a PCC could have a central role in communicating to parents/caregivers with CNS disorders the importance of the proper storage and management of medicines, with the aim of minimizing the risk of intoxication in children.

Considering the COVID-19 pandemic, for the three groups of substances that appear to be associated with COVID-19 cases, human exposure cases had two peaks [3]: one around 31 March to 3 April associated with increased exposures to cleaning/disinfectant agents (primarily bleaches), and one around July 15th, associated with a smaller peak in cleaning/disinfecting agents (primarily bleaches) and in hand sanitizer exposures. This trend was confirmed by a previous publication by our research group, where a higher frequency of suspected intoxications associated with both sanitizers/cleaners and bleaches was observed between the two study periods of January–April 2019 and January–April 2020 [7]. Considering the pediatric subjects analyzed in our study, from 2015 to 2019, the number of calls both from caregivers and healthcare professionals remained quite stable, as well as the ED visits or hospitalizations requested by clinical toxicologists. On the contrary, during the COVID-19 pandemic (first wave) experienced in 2020, an increase of calls from caregivers was observed. Accordingly, the number of calls received by healthcare professionals and the number of ED visits or hospitalizations requested by clinical toxicologists decreased, probably due to the social restrictions imposed by the pandemic emergency.

### Limitations and Strengths

The present study has several limitations. First of all, it is necessary to remember that our analysis is a retrospective investigation, and for this reason, data collection might be inaccurate, since some patients’ demographic and clinical variables were not always available. Moreover, we do not have a global overview of exposures and suspected intoxications in children, since they only come to healthcare professionals’ attention if suspected agents are perceived as dangerous by parents/caregivers. Thus, an underestimation of total exposures and suspected intoxications could not be completely excluded. Additionally, it is difficult to compare the different series, because some papers, both at a national or international level, include patients up to 16 or 18 years of age. Although our data consider exposures and suspected intoxications that mainly occurred in children in central Italy, this study is not representative of Italy as a whole.

The major strength of our study relies on the fact that a large cohort of children aged 0–14 were evaluated over a relatively long follow-up period. In fact, this allowed us to evaluate changing trends in consultations for exposures and suspected intoxications in children before and during the COVID-19 pandemic in Italy. The utilization of a local validated electronic database (ArchiCav), which includes all demographic and clinical information of all the subjects who came into contact with our toxicology unit and PCC, allowed us to perform a detailed and extensive epidemiological analysis. This approach, as already demonstrated in our previously published analysis [7,13,14,15,16], could represent a useful source of new scientific insights for clinical toxicologists into the management of phone call consultations in the general population, particularly in children.

## 5. Conclusions

Despite exposures and suspected intoxications being a relevant problem in children aged 0–14, our results confirmed the clinical characteristics of these potential life threatening occurrences, being comparable with those reported both at the Italian and international level by other colleagues. Furthermore, our results highlighted the need for public health authorities to program preventive interventions, which should involve the daily clinical practice of all healthcare professionals, including general physicians and community pharmacists.

Of course, it is necessary to identify shared national best practices for prevention of acute poisonings in childhood. Understanding which pediatric exposures and suspected intoxications require a clinical toxicologist management, the agents most frequently involved, and the circumstances is paramount for providing education for parents/caregivers and providers.

In the context of a venerable subset, such as the pediatric population, our results showed the utility of the PCC counselling in avoiding unnecessary visits to the ED, which is a relevant achievement, particularly in the time of COVID-19 pandemic, as well as the value of specialized clinical toxicologists in managing serious exposures.

## Figures and Tables

**Table 1:** Demographic and clinical characteristics (N = 11,996).

Demographic and Clinical Characteristics	0–11 Months N = 1220 (%)	1–5 Years N = 8854 (%)	6–11 Years N = 1451 (%)	>12 Years N = 471 (%)	*p*-Value
Sex					
Male	633 (51.89)	4779 (53.98)	855 (58.92)	222 (47.13)	<0.001
Female	587 (48.11)	4075 (46.02)	596 (41.08)	249 (52.87)	
Route of exposure					
Oral	1133 (92.87)	8279 (93.51)	1263 (87.04)	408 (86.62)	<0.001
Skin	15 (1.23)	220 (2.48)	64 (4.41)	24 (5.10)	
Ocular	16 (1.31)	215 (2.43)	33 (2.27)	10 (2.12)	
Inhalation	35 (2.87)	108 (1.22)	77 (5.31)	27 (5.73)	
Other route of exposure	21 (1.72)	32 (0.36)	14 (0.96)	2 (0.42)	
Qualification					
Caregiver	768 (62.95)	5050 (57.04)	745 (51.34)	140 (29.72)	<0.001
Healthcare professional	452 (37.05)	3804 (42.96)	706 (48.66)	331 (70.28)	
Location of exposure					
Home	1189 (97.46)	8656 (97.76)	1343 (92.56)	418 (88.75)	<0.001
Others	31 (2.54)	198 (2.24)	108 (7.44)	53 (11.25)	
Circumstances					
Involuntary intoxication	1220 (100)	8854 (100)	1445 (99.59)	362 (76.86)	<0.001
Domestic exposure	980	8333	1198	287	
Therapeutic error	231	511	242	74	
Adverse reactions	9	10	5	1	
Voluntary intoxications	-	-	6 (0.41)	109 (23.14)	
Suicide attempt	-	-	6	94	
Substance abuse	-	-	-	15	
Time from exposure					
Mean ± SD (hours)	5.75 ± 40.66	2.76 ± 17.68	5.20 ± 19.58	6.13 ± 30.09	0.997
<30 min	621 (50.90)	4388 (49.56)	563 (38.80)	134 (28.45)	<0.001
30–60 min	204 (16.72)	1776 (20.06)	247 (17.02)	79 (16.77)	
60 min–24 h	341 (27.95)	2447 (17.02)	555 (38.25)	232 (49.26)	
≥24 h	54 (4.43)	243 (2.74)	86 (5.93)	26 (5.52)	
Symptoms					
Absent	1067 (87.46)	7494 (84.64)	1054 (72.64)	271 (57.54)	<0.001
Gastrointestinal	77 (6.31)	755 (8.53)	190 (13.90)	78 (16.56)	
Neurologic	35 (2.87)	140 (1.58)	72 (4.96)	70 (14.86)	
Cutaneous	12 (0.98)	159 (1.80)	67 (4.62)	24 (5.10)	
Ocular	6 (0.49)	174 (1.97)	23 (1.59)	9 (1.91)	
Respiratory	10 (0.82)	92 (1.04)	31 (2.14)	13 (2.76)	
Cardiac	4 (0.33)	13 (0.15)	9 (0.62)	5 (1.06)	
Other	9 (0.74)	27 (0.30)	5 (0.34)	1 (0.21)	
Toxicologist advices					
Observation at home	908 (74.43)	6165 (69.63)	967 (66.64)	188 (39.92)	<0.001
ED visit for outpatients	89 (7.30)	795 (8.98)	118 (8.13)	55 (11.68)	
ED observation for inpatients	146 (11.97)	1318 (14.89)	224 (15.44)	117 (24.84)	
Hospitalisation	77 (6.31)	576 (6.51)	142 (9.79)	111 (23.57)	
Prescribed therapies					
No	989 (81.07)	6433 (72.66)	997 (68.71)	219 (46.50)	<0.001
Yes	231 (18.93)	2421 (27.34)	454 (31.29)	252 (53.50)	
Symptomatic therapies	190	1933	358	170	
Decontamination	31	444	69	63	
Antidotal therapies	10	44	27	19	

ED: emergency department; SD: standard deviation.

**Table 2:** Description of toxic agents according to age classes (N = 11,996).

	0–11 Months N = 1220 (%)	1–5 Years N = 8854 (%)	6–11 Years N = 1451 (%)	>12 Years N = 471 (%)
Number of toxic agents				
One	1211 (99.26)	8754 (98.87)	1434 (98.83)	434 (92.14)
More than one	9 (0.74)	100 (1.13)	17 (1.17)	37 (7.86)
Toxic agents				
Non-pharmacological agents	854 (70.00)	6144 (69.39)	952 (65.61)	256 (54.35)
Cleaners	160	1947	171	55
Other household products	126	951	190	41
Toys	105	844	145	18
Cosmetics	90	817	52	19
Plant poisons	164	589	95	14
Pesticides	120	449	66	8
Foods	32	193	85	23
Caustics	12	219	49	16
Animal poisons	12	80	59	24
Substances of abuse	16	44	9	28
Carbon monoxide or other gasses	17	33	34	12
Pharmacological agents ^§^	366 (30.00)	2707 (30.57)	499 (34.39)	213 (45.22)
Analgesics (ATC N02)	63	269	43	32
Antibacterials for systemic use (ATC J01)	30	182	49	15
NSAIDs and antirheumatic products (ATC M01)	8	209	38	22
Psycholeptics (ATC N05)	11	118	51	63
Thyroid therapy (ATC H03)	7	170	16	3
Psychoanaleptics (ATC N06)	9	98	37	24
Drugs for obstructive airway diseases (ATC R03)	13	120	17	0
Sex hormones and modulators of the genital system (ATC G03)	0	116	11	1
Antihistamines for systemic use (ATC R06)	9	81	19	5
Agents acting on the renin–angiotensin system (ATC C09)	5	98	6	7
Antiepileptics (ATC N03)	4	59	29	23
Nasal preparations (ATC R01)	19	66	9	2
Mineral supplements (ATC A12)	27	52	9	0
Cough and cold preparations (ATC R05)	4	61	11	4
Beta blocking agents (ATC C07)	2	57	5	6
Complementary and alternative medicine	36 (2.95)	238 (2.68)	44 (3.03)	8 (1.69)
Dietary supplements	25	144	32	2
Phytotherapy	9	56	7	4
Homeopathy	2	38	5	2

ATC: anatomical, therapeutic, chemical classification system; NSAIDs: non-steroidal anti-inflammatory drugs. The distribution of variables was statistically different among age groups for the number of toxic agents and for toxic agents’ description. ^§^ Only the first 15 most frequently reported pharmacological agents are depicted in the table.

**Table 3:** Risk of ED visit or hospitalization according to demographic and clinical characteristics.

	Crude Odds Ratio (95% Confidence Interval)	Adjusted Odds Ratio (95% Confidence Interval)
Sex		
Male	1	1
Female	0.92 (0.86–1.00)	0.88 (0.81–0.95)
Age		
0–11 months	1	1
1–5 years	1.27 (1.11–1.45)	1.27 (1.10–1.45)
6–11 years	1.46 (1.23–1.72)	1.35 (1.14–1.60)
>12 years	4.38 (3.50–5.48)	2.52 (1.97–3.22)
Number of toxic agents		
One	1	1
More than one	5.53 (3.93–7.78)	3.79 (2.65–5.42)
Circumstances		
Involuntary intoxication	1	1
Voluntary intoxications	51.31 (20.94–125.74)	29.18 (11.76–72.38)
Toxic agents		
Non-pharmacological agents	1	1
Pharmacological agents	1.40 (1.29–1.52)	1.34 (1.23–1.46)
Pharmacological and non-pharmacological agents	9.76 (1.09–87.39)	2.03 (0.20–20.13)
Route of exposure		
Other route of exposure	1	1
Oral	0.53 (0.33–0.86)	0.57 (0.35–0.93)
Skin	0.60 (0.35–1.01)	0.68 (0.40–1.16)
Ocular	0.91 (0.54–1.55)	1.07 (0.63–1.84)
Inhalation	1.74 (1.02–2.98)	1.87 (1.09–3.23)

**Table 4:** Risk of ED visit or hospitalization according to the most frequently reported pharmacological drug classes.

	Crude Odds Ratio (95% Confidence Interval)	Adjusted Odds Ratio (95% Confidence Interval)
Analgesics (ATC N02)	1.07 (0.87–1.33)	1.05 (0.84–1.30)
NSAIDs and antirheumatics (ATC M01)	0.65 (0.49–0.86)	0.65 (0.49–0.86)
Antibacterials for systemic use (ATC J01)	0.41 (0.30–0.55)	0.37 (0.27–0.50)
Psycholeptics (ATC N05)	4.81 (3.56–6.50)	4.25 (3.13–5.78)
Thyroid therapy (ATC H03)	0.62 (0.45–0.86)	0.67 (0.48–0.93)
Psychoanaleptics (ATC N06)	3.88 (2.75–5.47)	3.68 (2.60–5.20)
Drugs for obstructive airway diseases (ATC R03)	0.44 (0.29–0.67)	0.46 (0.30–0.69)
Sex hormones (ATC G03)	0.42 (0.27–0.65)	0.45 (0.29–0.71)
Agents acting on the renin–angiotensin system (ATC C09)	3.85 (2.51–5.89)	4.13 (2.69–6.34)
Antiepileptics (ATC N03)	2.36 (1.61–3.47)	2.08 (1.40–3.07)
Antihistamines for systemic use (ATC R06)	1.92 (1.30–2.82)	1.92 (1.30–2.84)
Nasal preparations (ATC R01)	0.41 (0.24–0.69)	0.42 (0.25–0.71)
Mineral supplements (ATC A12)	0.82 (0.52–1.30)	0.89 (0.56–1.40)
Cough and cold preparations (ATC R05)	0.80 (0.50–1.30)	0.80 (0.49–1.29)
Beta blocking agents (ATC C07)	10.23 (5.02–20.84)	10.99 (5.38–22.44)

ATC: anatomical therapeutic chemical; NSAIDs: non-steroidal anti-inflammatory drugs.

## Data Availability

Data that support the findings of this study are available upon reasonable request from the corresponding author, A.V.

## Supplementary Materials

The following supporting information can be downloaded at: https://www.mdpi.com/article/10.3390/jcm12010352/s1, Table S1: Time elapsed between the exposure and the calling to the poison control center; Table S2: Classification of toxic agents according to the qualification of the caller.
