# Supplementary material for: Exposures and Suspected Intoxications to Pharmacological and Non-Pharmacological Agents in Children Aged 0–14 Years: Real-World Data from an Italian Reference Poison Control Centre
Source: J Clin Med. 2023 Jan 2;12(1):352. doi: 10.3390/jcm12010352 (PMC9820854; doi:10.3390/jcm12010352)
Supplement: Supplementary file 1 [file jcm-12-00352-s001.zip › jcm-2115713-supplementary.pdf]

Supplementary Tables

Table S1. Time elapsed between the exposure and the calling to the poison control centre.

	Hours (mean±SD)	p-value
Toxic agents		
Non-pharmacological agents	3.30±20.30	0.998
Pharmacological agents	3.92±25.17	
Pharmacological and non-pharmacological agents	1.20±1.04	
Qualification		
Caregiver	3.07±19.12	0.992
Healthcare professional	4.03±25.02	

SD: standard deviation.

Table S2. Classification of toxic agents according to the qualification of caller.

	Caregiver N=6703 (%)	Healthcare professional N=5293 (%)	p-value
Toxic agents			
Non-pharmacological agents	4799 (71.59)	3407 (64.37)	<0.001
Pharmacological agents	1903 (28.39)	1882 (35.56)	
Pharmacological and non-pharmacological agents	1 (0.01)	4 (0.08)	
